# Ligand Binding and Signaling of Dendritic Cell Immunoreceptor (DCIR) Is Modulated by the Glycosylation of the Carbohydrate Recognition Domain

**DOI:** 10.1371/journal.pone.0066266

**Published:** 2013-06-11

**Authors:** Karien Bloem, Ilona M. Vuist, Arend-Jan van der Plas, Léon M. J. Knippels, Johan Garssen, Juan J. García-Vallejo, Sandra J. van Vliet, Yvette van Kooyk

**Affiliations:** 1 Department of Molecular Cell Biology and Immunology, VU University Medical Center, Amsterdam, The Netherlands; 2 Danone Research, Centre for Specialized Nutrition, Wageningen, The Netherlands; 3 Department of Pharmacology and Pathophysiology, Utrecht Institute for Pharmaceutical Sciences (UIPS), Utrecht University, Utrecht, The Netherlands; Bangor University, United Kingdom

## Abstract

C-type lectins are innate receptors expressed on antigen-presenting cells that are involved in the recognition of glycosylated pathogens and self-glycoproteins. Upon ligand binding, internalization and/or signaling often occur. Little is known on the glycan specificity and ligands of the Dendritic Cell Immunoreceptor (DCIR), the only classical C-type lectin that contains an intracellular immunoreceptor tyrosine-based inhibitory motif (ITIM). Here we show that purified DCIR binds the glycan structures Lewis^b^ and Man_3_. Interestingly, binding could not be detected when DCIR was expressed on cells. Since DCIR has an *N*-glycosylation site inside its carbohydrate recognition domain (CRD), we investigated the effect of this glycan in ligand recognition. Removing or truncating the glycans present on purified DCIR increased the affinity for DCIR-binding glycans. Nevertheless, altering the glycosylation status of the DCIR expressing cell or mutating the *N*-glycosylation site of DCIR itself did not increase glycan binding. In contrast, *cis* and *trans* interactions with glycans induced DCIR mediated signaling, resulting in a decreased phosphorylation of the ITIM sequence. These results show that glycan binding to DCIR is influenced by the glycosylation of the CRD region in DCIR and that interaction with its ligands result in signaling via its ITIM motif.

## Introduction

C-type lectin receptors (CLRs) are glycan binding receptors present on the surface of immune cells. CLRs are involved in the recognition of pathogens; however self-ligands for CLRs have been described as well [1]. Most CLRs expressed on dendritic cells (DCs), like dendritic cell-specific intercellular adhesion molecule 3 (ICAM-3) grabbing non-integrin (DC-SIGN) [2], macrophage galactose-type lectin (MGL) [3] and the mannose receptor (MR) [4], can function as antigen uptake receptors. In addition, signaling or modulation of Toll-like receptor (TLR) responses has also been described for some CLRs [5]. The CLR dendritic cell immunoreceptor (DCIR) is expressed on a variety of immune cells, such as DCs, B-cells and monocytes [6]. DCIR is the only classical CLR with an immunoreceptor tyrosine-based inhibitory motif (ITIM) in its cytoplasmic tail. ITIMs can interact with Src Homology 2 (SH2) domain containing protein tyrosine phosphatase (SHP) 1 or 2 or the SH2 domain containing inositol 5-phosphatase (SHIP). These phosphatases are able to dephosphorylate signaling molecules [7]. The ITIM in DCIR is able to recruit SHP-1 and SHP-2, which requires phosphorylation of the DCIR ITIM [8], [9]. Binding to SHIP has not been observed [9].

The role of DCIR in regulating immune responses has been investigated in *dcir* ^−/−^ mice. These mice develop spontaneous autoimmunity at later age [10]. Furthermore, the response to collagen-induced arthritis was increased in *dcir* ^−/−^ mice. Therefore, DCIR appears to have a role in keeping the immune system in a quiescent state. This regulatory role of DCIR corresponds with findings that SNPs in the human DCIR locus are associated with the development of the auto-immune disease rheumatoid arthritis [11], [12].

The signaling properties of DCIR have been investigated in a B cell line lacking Fc receptors that was transduced with a chimeric protein containing the cytoplasmic tail of the mouse homologue *dcir1* coupled to the extracellular part of the FcγRIIB receptor. After simultaneous activation of the B cell receptor, signaling by the chimeric DCIR-FcγRIIB receptor inhibited the release of intracellular calcium [13]. These effects were dependent on the ITIM in DCIR, as inhibition of intracellular calcium release was not observed in cells transduced with DCIR containing a non-functional ITIM. This blockade in the calcium release correlated with dephosporylation of several signaling proteins, as total protein phosphorylation seen after B cell receptor stimulation was decreased in cells transduced with the DCIR-FcγRIIB chimeric receptor as well.

In both plasmacytoid DCs and monocyte-derived DCs (moDCs) triggering of DCIR with a monoclonal antibody modulated TLR9 or TLR7/8 responses, respectively. A decrease in cytokines (IFNα and TNFα for pDCs and IL-12 and TNFα for moDCs) was observed when both TLR and DCIR were simultaneously triggered [14], [15]. However, which pathway is elicited after DCIR stimulation, leading to inhibition of TLR signaling, remains unsolved.

In order to gain more insight in the signaling function of DCIR it is important to elucidate the glycan specificity of DCIR and DCIR binding ligands. CLRs can be divided in two groups based on their glycan binding specificity, which is dictated by an amino acid sequence triplet in the carbohydrate recognition domain (CRD) [16]. The galactose-type lectins, like MGL, have a QPD motif present in their CRD [17], [18], whereas fucose/mannose binding lectins, such as DC-SIGN [19] and MR [20], contain an EPN motif. Instead of an EPN motif, the putative carbohydrate binding site of DCIR contains the unusual sequence EPS [6]. Since this EPS motif only differs in one amino acid compared to the fucose and mannose binding EPN motif, DCIR binding to fucose and mannose glycans has been hypothesized. This specificity has been confirmed by Lee *et al*. [21]. However, binding of purified human DCIR to sulfated LacNAc and Lac, and biantennary *N*-glycans has also been reported [22].

Glycan binding to DCIR expressed on cells has not been reported yet. Therefore, details on the biology of glycan-ligand interaction with cellular DCIR are still lacking. We hypothesize that the ligand-binding site of DCIR expressed on cells is occupied. This blocking of the carbohydrate-binding site may be caused by the potential interaction with *cis* ligands present on adjacent glycoproteins, similar to the concept of masking described for siglecs [23]. Siglecs are known for their specificity for sialic acids, a common terminal modification of glycans. Sialic acids expressed on adjacent proteins have been shown to mask siglecs. These sialic acids can be removed by sialidase treatment leading to unmasking and enhanced binding of *trans* ligands by siglecs. Alternatively, glycans present on DCIR itself could hinder or occupy its carbohydrate-binding site. Interestingly, the only predicted *N*-glycosylation site for DCIR is located within the putative CRD [6]. *N*-glycosylation of DCIR has been demonstrated in neutrophils [24], whereby *N*-glycosidase treatment of immunoprecipitated DCIR resulted in a decrease in the apparent molecular weight. However, the role of this *N*-glycan in regulating DCIR ligand binding has never been addressed.

We therefore investigated whether glycan binding of DCIR was affected by the DCIR glycosylation status. Using a DCIR-Fc chimeric protein produced in Chinese hamster ovary (CHO) Lec8 cells [25] we found an increased binding to Lewis^b^ and Man_3_. A DCIR-Fc mutant lacking the *N*-linked glycosylation site also showed enhanced glycan binding. Surprisingly, no glycan binding of DCIR expressed on CHO Lec8 cells or DCIR lacking its *N*-glycosylation site expressed on CHO or CHO Lec8 cells could be observed. Interestingly, although no adhesive interaction of the glycans was observed, the phosphorylation status of DCIR expressed on the CHO Lec8 cells was greatly affected upon ligand stimulation, indicating that absence of DCIR binding glycans on the cells renders DCIR sensitive for glycan-induced signaling.

## Materials and Methods

### Antibodies, Fc Chimeric Proteins, Lectins And Glycans

DC-SIGN-Fc was produced as described previously [26]. DCIR-Fc consists of the extracellular domains of DCIR (amino acid residues 208–689) fused at the C terminus to the Fc domain of human IgG1 in the Sig-pIgG1-Fc vector [27]. The DCIR glycosylation mutant Fc construct (DCIR_N185Q_-Fc) was made with the use of QuikChange II Site-Directed Mutagenesis Kit (Stratagene). Sequences were tested for correct insertion of the mutation (BaseClear). DCIR-Fc and DCIR_N185Q_-Fc were produced by co-transfection of the DCIR-Fc or DCIR_N185Q_-Fc vector with pEE14 in CHO cells. DCIR-Fc vector was co-transfected with pGK HYG in CHO Lec8 cells for the production of DCIR-Fc Lec8. DC-SIGN-Fc, DCIR-Fc, DCIR-Fc Lec8 and DCIR_N185Q_-Fc were purified with Hi Trap Protein A HP columns (GE Healthcare). DCIR expressing cells were made with the pRRL-cPPT-CMV-X2-PRE-SIN-IRES-eGFP vector [28] in which the IRES-eGFP was replaced with the complete open reading frame of DCIR. The glycosylation mutant DCIR_N185Q_ was made using the QuikChange II Site-Directed Mutagenesis Kit (Stratagene). Sequences were tested for correct insertion of the mutation (BaseClear). Antibodies used are α-DCIR 111F8.04 (Dendritics), α-DC-SIGN AZN-D1 [29], α-Langerin 10E2 [30], α-phosporylated tyrosine PY20 (Millipore), goat anti-mouse IgG Alexa Fluor 488 F(ab′)_2_ fragment (Invitrogen), goat-anti-human Fc-PO (Jackson Immunoresearch), strepatavidin-PO (Invitrogen), goat-anti-human Fc (Jackson), goat-anti-human Fc-biotin (Jackson), streptavidin-Alexa Fluor 647 and 488 (Invitrogen) and IRDye goat-α-mouse IgG 800CW (Li-Cor). Lectins used are: ConA, MAA-II, SNA, PNA, SBA (Vector Labs) and HPA (Sigma Aldrich), all biotin labeled. Glycans used were biotin labeled polyacrylamide (PAA) conjugates (Lectinity), except for Mannotriose (Dextra), which was conjugated to BSA (Man_3_BSA) and biotin labeled in house. Glycan and Fc coated beads were made as described previously [31]. For Fc beads 10 µg of biotin labeled goat-anti-human-Fc was added to fluorescent-streptavidin coupled beads, where after 1 µg of Fc construct was coupled to these beads. Human IgG1 kappa (Serotec) was used as a control Fc protein. For the production of the glycan beads 4 µg of biotin labeled glycans were coupled to the fluorescent-streptavidin coupled beads.

### Cells

CHO (CHO-K1 from ATCC) and CHO Lec8 cells (a kind gift from Prof. dr. P. Stanley [32]) were transduced as described previously [33], [34] and cultured in RPMI1640 (Invitrogen) supplemented with 1000 Units/ml penicillin/streptomycin (Lonza), 2 mM glutamine (Lonza) and 10% FBS (BioWhittaker). CHO-DC-SIGN cells were described previously [35] and cultured in the presence of 1 mg/ml Geneticin (Invitrogen). Fc-protein producing cell lines were either cultured in RPMI supplemented with 1000 Units/ml penicillin/streptomycin, 2 mM glutamine, 60 µg/ml glutamic acid, 60 µg/ml asparagine, 7 µg/ml adenosine, 7 µg/ml guanosine, 7 µg/ml cytidine, 7 µg/ml uridine and 2.4 µg/ml thymidine (Sigma Aldrich), 1 mM MEM non-essentials amino acids, 1 mM Sodium Pyruvate MEM (Invitrogen) and 10% dialyzed FBS (Invitrogen) for CHO-DCIR-Fc, CHO-DCIR_N185Q_-Fc and CHO-DC-SIGN-Fc or in RPMI supplemented with 1000 Units/ml penicillin/streptomycin, 2 mM glutamine, 10% FBS and 500 µg/ml hygromycin (Invitrogen) for CHO Lec8 DCIR-Fc, to select for Fc producing cells.

### Clr-Fc Binding Assay

Goat-anti-human Fc (4 µg/ml) coated ELISA plates (Maxisorp, Nunc) were blocked with 1% ELISA grade BSA (Fraction V, Fatty acid free; Calbiochem) in TSM (Tris Saline Magnesium buffer: 20 mM Tris, pH 7.4, 150 mM NaCl, 1 mM CaCl_2_ and 2 mM MgCl_2_) buffer. 1 µg/ml of the CLR-Fc construct was added to the plates. Subsequently, glycans were added at 5 µg/ml or at indicated concentrations in TSM/BSA in the presence or absence of the Ca^2+^ chelator EGTA (10 mM), a well-known blocker of CLR function, for at least 2 hours at room temperature. After extensive washing with TSM/0.05% Tween (Sigma Aldrich), streptavidin-PO (Invitrogen) was added and the reaction was developed with 3,3′,5,5′-tetramethylbenzidine (TMB) as a substrate (Sigma Aldrich).

### Glycan Binding Assay

5 µg/ml of glycan was coated to the ELISA plate in coating buffer (50 mM Na_2_CO_3_, pH 9.7) overnight at room temperature. After blocking with TSM/1% BSA, 50 µg/ml of the different DCIR-Fc constructs were added to the ELISA plate for at least 2 hours at room temperature. After extensive washing with TSM/0.05% Tween, 0.27 µg/ml goat-anti-human Fc-PO (Jackson Immunoresearch) in TSM/0.05% Tween was added and the reaction was developed with TMB as a substrate.

### Flow Cytometry

Expression of DCIR or DC-SIGN on cell lines was determined by staining with 5 µg/ml of α-DCIR or α-DC-SIGN respectively and with 5 µg/ml Alexa Fluor 488 F(ab′)_2_ fragment of goat anti-mouse IgG as a secondary antibody, and analyzed by flow cytometry (FACScan, BD biosciences). Binding of fluorescently labeled glycan beads (40 beads/cell), in the presence or absence of EGTA, to DCIR and DC-SIGN expressing cell lines was measured after 45 minutes incubation at 37°C in TSM/0.5% BSA (Fraction V; Roche). To test the binding of fluorescent glycan beads and biotin labeled neoglycoconjugates, the different DCIR expressing cell lines were incubated in serum free medium 1.5 hours before performing the experiment. Binding of biotin labeled neoglycoconjugates to DCIR and DC-SIGN expressing cell lines was performed by adding 10 µg/ml neoglycoconjugates pre-incubated with 2.5 µg/ml streptavidin-Alexa Fluor 647 in TSM/0.5% BSA for 2 hours at 37°C. Binding of glycan beads and neoglycoconjugates was analyzed by flow cytometry.

### Immunoprecipitation and Western Blot

Cells were washed with PBS and incubated in serum free medium for 1.5 hour. 1 mM sodium orthovanadate (New England Biolabs) was added for 30 minutes. For stimulation experiments cells were collected in 1 ml TSM after the serum free incubation period. 100 µg/ml of Lewis^b^-PAA was added for 5 minutes at 37°C, thereafter 1 mM of sodium orthovanadate was added for 30 minutes at 37°C. Cells were lysed in lysis buffer (1% Triton X-100, 1% sodium deoxycholate, 0.1% SDS, 150 mM NaCl, 50 mM Tris-HCl pH 7.2, 1 mM CaCl_2_, 1 mM MgCl_2_ and complete EDTA free protease inhibitor cocktail (Roche)) in the presence of 1 mM sodium orthovanadate. DCIR was immunoprecipitated with 5 µg α-DCIR 111F8.04 coupled to protein A Sepharose (Amersham Biosciences) and 5 µg α-Langerin 10E2 coupled to protein A Sepharose was used as isotype control. Immune precipitates were resolved by SDS-PAGE (12% polyacrylamide gel). Proteins were transferred to a nitrocellulose membrane (Whatman) and stained with either 3 µg/ml α-DCIR or 1 µg/ml α-phosporylated tyrosine (PY20). Antibodies were detected with the use of IRDye goat-α-mouse IgG 800CW (LI-COR). Signal intensities were analyzed using the Odyssey Infrared Imaging System (LI-COR).

## Results

### DCIR binds to Lewis^b^ and Man_3_

**Figure 1:**
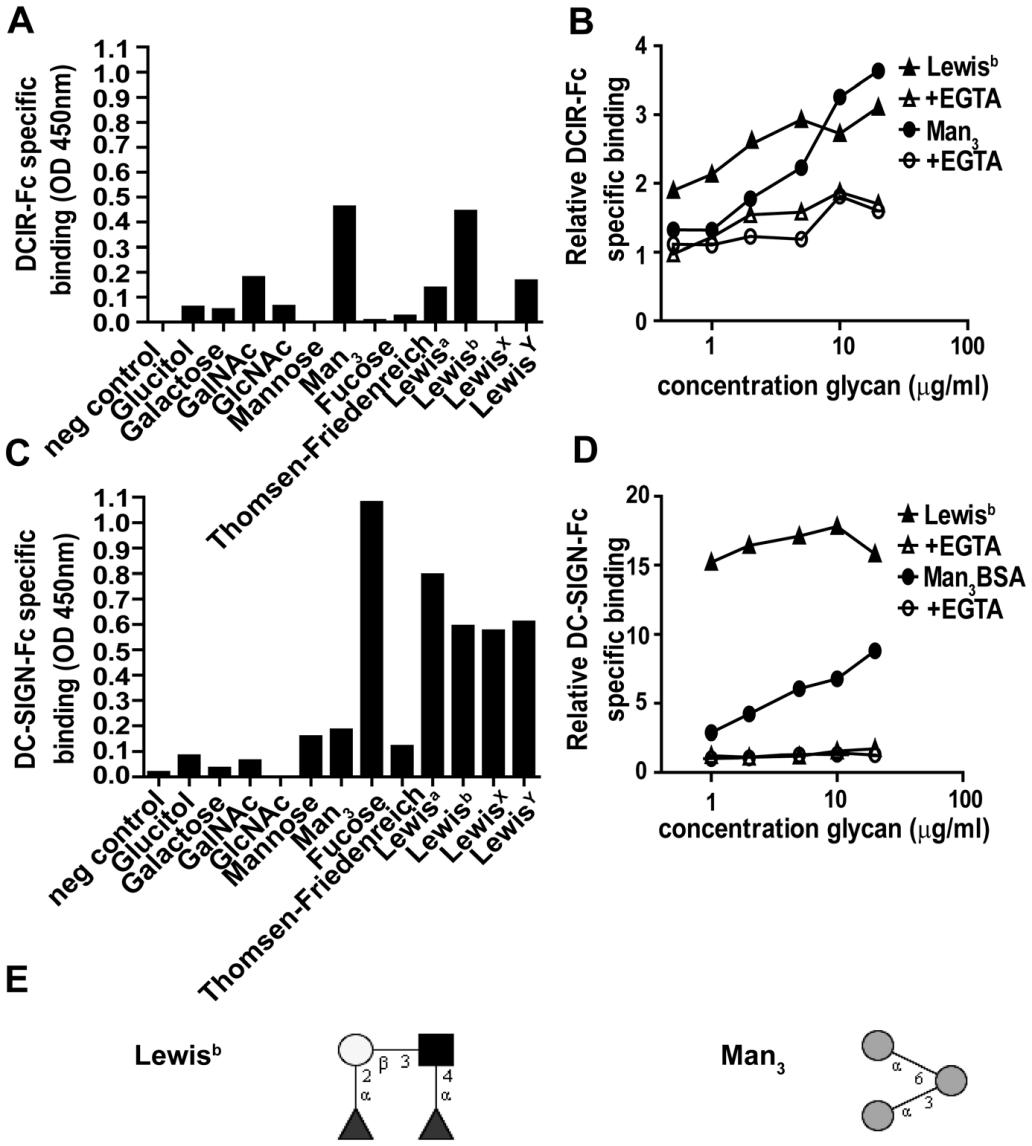
DCIR-Fc specifically binds Lewis^b^ and Man_3_ glycans. (A/C) DCIR-Fc binding to Lewis^b^ and Man_3_. Different biotin-labeled neoglycoconjugates were added at a concentration of 5 µg/ml to immobilized DCIR-Fc-(A) and DC-SIGN-Fc-(C). Depicted is Fc-specific binding with the EGTA control subtracted. One representative experiment out of 5 is shown. (B/D) Ligand binding of DCIR-Fc and DC-SIGN-Fc is concentration dependent. A concentration range of Man_3_ and Lewis^b^ was added to immobilized DCIR-Fc- (B) and DC-SIGN-Fc (D). Depicted is the relative binding compared to the BSA coated control. One representative experiment out of 5 is shown. (E) Glycan structures of Man_3_ and Lewis^b^ were drawn using the GlycoWorkbench software suite [53], [54]. White circle represents galactose, grey circle represents mannose, grey square represents *N*-acetylglucosamine (GlcNAc) and the grey triangle represents fucose.

Although binding of purified DCIR to fucose, mannose [21], sulfated-LacNAc/Lac and biantennary *N*-glycans [22] has been demonstrated, binding of these structures has never been confirmed using cell-based assays. This is important, since oligomerization of receptors or post-translational modifications have a great influence on the glycan binding activity of CLRs. We therefore compared the glycan binding specificity of purified recombinant DCIR (DCIR-Fc) and cellular DCIR. We first generated a DCIR-Fc chimeric construct, in which the CRD of DCIR was fused to the Fc tail of human IgG and used this DCIR-Fc construct in our CLR binding assay to search for new ligands for DCIR. We observed CLR-specific binding of Man_3_ and Lewis^b^ to immobilized DCIR-Fc (Figure 1A). Significant binding could not be observed for other fucose containing glycans, like Lewis^a^. The binding characteristics of DCIR were investigated using a titration of glycans. Lewis^b^ binding to DCIR-Fc reached a plateau already at 5 µg/ml, while Man_3_ did not even reach a plateau at 20 µg/ml (Figure 1B). DCIR-Fc binding in the presence of EGTA was negligible, confirming DCIR as classical Ca^2+^-dependent glycan binding CLR. To compare the glycan specificity of DCIR with that of DC-SIGN, which shares the ability to bind Lewis^b^ and Man_3_, we investigated glycan binding to immobilized DC-SIGN-Fc (Figure 1C-D). In addition to Lewis^b^ and Man_3_, DC-SIGN binding to other fucose containing glycans was observed as well, corresponding to the broader glycan specificity of DC-SIGN [19]. Glycan titration revealed DC-SIGN binding even at low glycan concentrations. However, at lower glycan concentrations both DC-SIGN-Fc and DCIR-Fc displayed enhanced binding of Lewis^b^ compared to Man_3_. Glycan structures of Man_3_ and Lewis^b^ are depicted in Figure 1E.

### Cellular DCIR does not Bind Glycans

**Figure 2:**
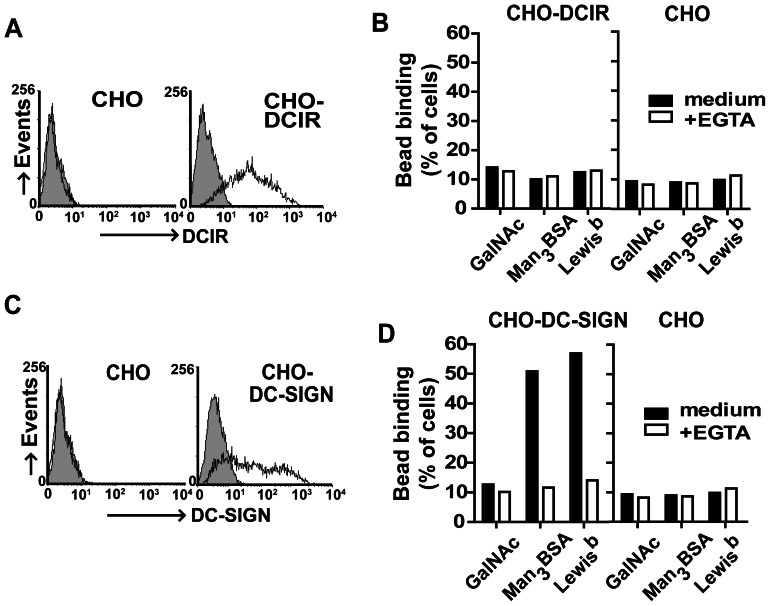
Cellular expressed DCIR does not interact with Lewis^b^ and Man_3_. (A) DCIR expression on CHO-DCIR cells was determined by flow cytometry. Filled histogram represents isotype control and black line indicates the DCIR expression. (B) Ligand binding to cellular expressed DCIR could not be observed. Binding of fluorescent labeled glycan coated beads to CHO-DCIR was analyzed by flow cytometry. One representative experiment out of 6 is shown. (C) DC-SIGN expression on CHO-DC-SIGN cells was determined by flow cytometry. Filled histogram represents isotype control and black line indicates the DC-SIGN expression. (D) Binding of fluorescent labeled glycan beads to CHO-DC-SIGN was measured by flow cytometry. One representative experiment out of 6 is shown.

To confirm the DCIR binding of Man_3_ and Lewis^b^ in a cell-based assay, we made DCIR-expressing CHO cell lines. Figure 2A demonstrates that the DCIR-transduced cell line expressed high levels of DCIR. Fluorescent beads coated with Man_3_ and Lewis^b^ were analyzed by flow cytometry for binding to the DCIR expressing cells. Surprisingly, significant binding of any of the beads could not be detected (Figure 2B). CHO-DCIR binding to glycan beads varied between 1–15% in the independent experiments, which could not be significantly reduced in the presence of EGTA. In contrast, DC-SIGN expressing CHO cells showed strong binding of the Man_3_ and Lewis^b^ coated beads (Figure 2C&2D), varying between 40 and 80% in independent experiments, confirming earlier research. Glycan bead binding was not observed to the parental CHO cells.

### Cell Glycosylation May Affect Dcir Binding Activity

**Figure 3:**
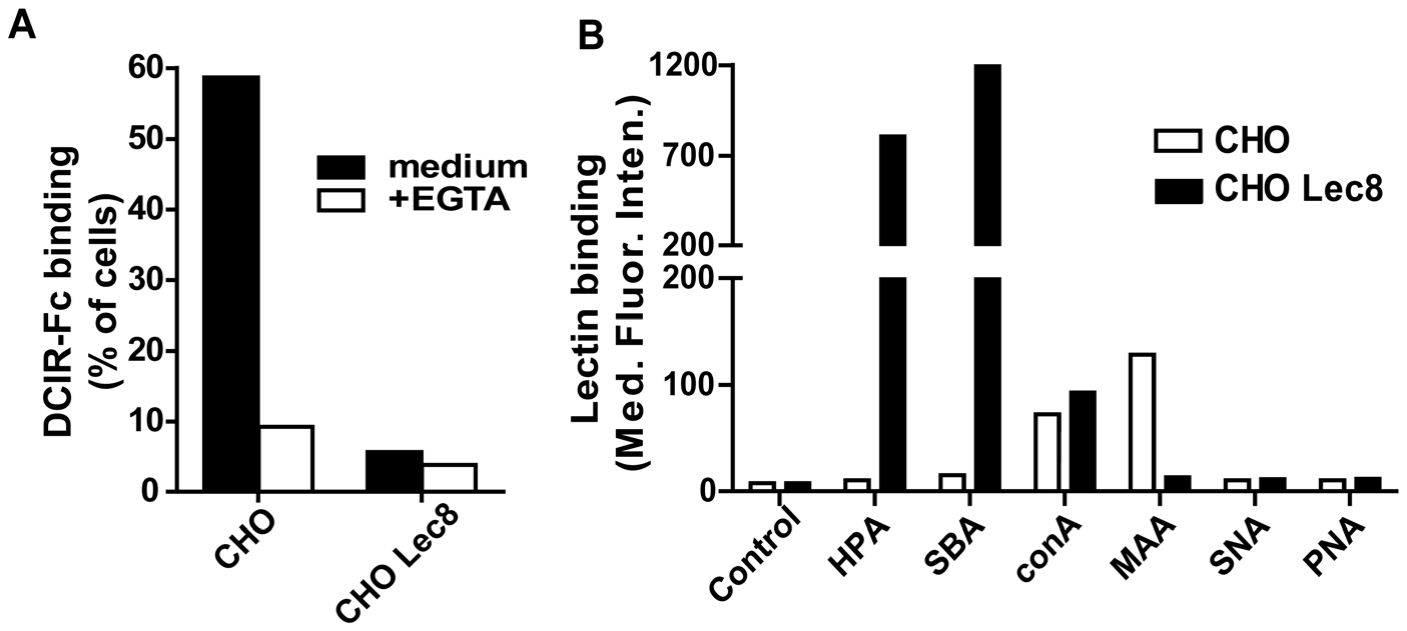
CHO Lec8 cells lack DCIR binding glycans. (A) DCIR binding to CHO cells. Binding of DCIR-Fc beads to CHO and CHO Lec8 cells was measured by flow cytometry. Percentage of cells that bound the CLR-Fc beads is depicted. One representative experiment out of 6 is shown. (B) Glycan profiling of CHO and CHO Lec8 cells reveals truncated glycans present on CHO Lec8 cells. Binding of lectins was measured by flow cytometry. Median fluorescence intensity is depicted. One representative experiment out of 5 is shown.

Because binding of cellular DCIR to Lewis^b^ and Man_3_ was not observed, we speculated that the carbohydrate-binding site might be occupied or hindered in CHO cells. Masking of binding sites has previously been shown for siglecs [23]. We used a DCIR-Fc-coated beads binding assay to evaluate the presence of DCIR binding glycans on CHO cells. DCIR-Fc-coated beads strongly bound parental CHO cells (Figure 3A). We then tested CHO Lec8 cells, that synthesize truncated complex *N*-glycans with terminal β-linked GlcNAc residues [36], due to a lack of a functional UDP-Gal transporter [25]. As expected, binding of the DCIR-Fc-coated beads to CHO Lec8 cells could not be observed (Figure 3A). Glycan profiles of CHO and CHO Lec8 cells have been extensively studied and published previously [36]–[38]. Our lectin staining confirms the reported glycosylation patterns on CHO and CHO Lec8 and demonstrates the presence of truncated glycans on CHO lec8 cells (Figure 3B). Due to presence of these truncated glycans, CHO Lec8 cells lack expression of DCIR binding glycans.

### Changes in the Glycosylation of Dcir Result in Enhanced Ligand Binding

**Figure 4:**
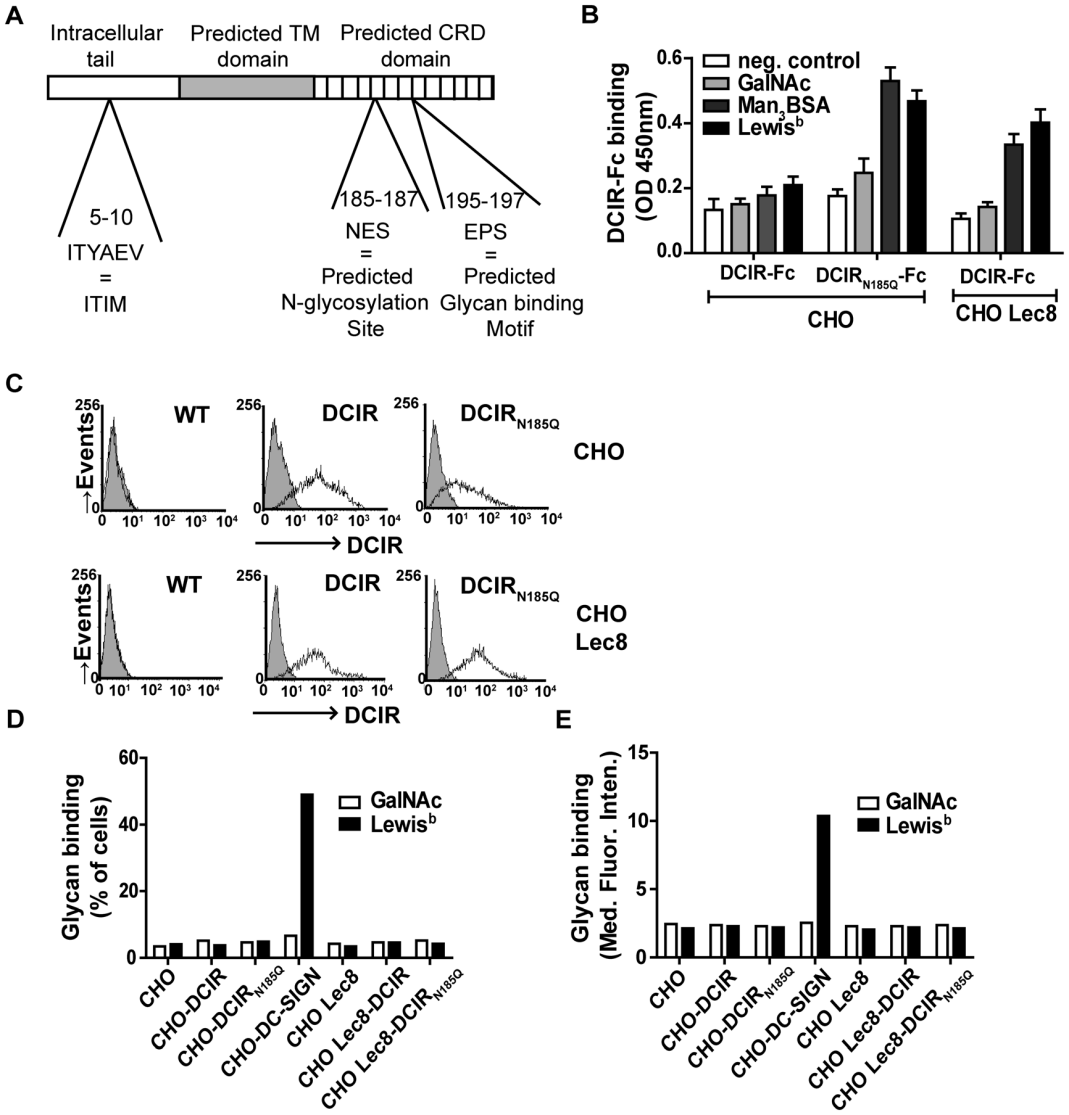
Glycosylation of the CRD of DCIR modulates its binding to glycan structures. (A) Schematic overview of the *N*-glycosylation site inside the putative CRD of DCIR. NES is the only predicted *N*-glycosylation site of DCIR. The EPS motif is the predicted glycan binding motif. Adapted from Bates *et al*. [(6)]. (B) Increased binding to DCIR ligands in the presence of truncated or complete absence of glycans on the DCIR-Fc construct. Binding of wild-type and the glycosylation mutant DCIR-Fc to immobilized neoglycoconjugates was measured in a glycan binding assay. Average DCIR-Fc binding ± SEM was calculated from 4 independent experiments. (C) DCIR expression on DCIR transduced cell lines was determined by flow cytometry. Filled histogram represents isotype control and black line indicates the DCIR expression. (D) Binding of glycan beads to cellular DCIR could not be detected. Binding of fluorescent labeled glycan beads to the different DCIR expressing cell lines was measured by flow cytometry in the absence of serum. As a control binding of the fluorescent labeled glycan beads to CHO-DC-SIGN was measured. One representative experiment out of 3 is shown. (E) Glycan binding of PAA-glycans to cellular DCIR could not be detected. Binding of biotinylated PAA-glycans pre-incubated with streptavidin-Alexa Fluor 647 to the different DCIR and DC-SIGN expressing cell lines was measured by flow cytometry after 2 hours incubation at 37°C. Binding of biotinylated PAA-glycans to CHO-DC-SIGN served as a positive control. One representative experiment out of 3 is shown.

Strikingly, there is a predicted *N*-glycosylation sequence (N_185_) within the putative CRD of DCIR, only a few amino acids away from the expected carbohydrate-binding site of DCIR (195–197) (Figure 4A). Glycans present in this region of DCIR might influence the binding of the DCIR molecule to DCIR binding ligands, either by *cis* interactions or through steric hindrance, in addition to the masking of DCIR in CHO cells. Since CHO Lec8 cells lack DCIR binding glycans, we hypothesized that production of DCIR-Fc by these cells would expose truncated glycans in its *N*-glycosylation site that would not interfere with the glycan binding site. In addition, we generated a glycosylation mutant of DCIR (DCIR_N185Q_-Fc) that lacks the only *N*-glycosylation site in the CRD of DCIR. Differences in glycosylation of the wild-type and glycosylation mutant DCIR-Fc construct were investigated by comparing their apparent molecular weight analyzed by SDS-PAGE (Figure S1). A decrease in apparent molecular weight was observed for DCIR_N185Q_-Fc compared to wild-type DCIR-Fc, indicating more extensive glycosylation of the wild-type DCIR-Fc construct.

Whereas wild-type DCIR-Fc produced in CHO cells showed negligible binding to immobilized glycans, the glycosylation mutant construct produced in wild-type CHO cells (DCIR_N185Q_-Fc) and the DCIR-Fc construct produced in CHO Lec8 cells (DCIR-Fc Lec8) strongly interacted with immobilized Lewis^b^ and Man_3_ (Figure 4B). These results indicate that binding of DCIR-Fc to immobilized glycans could be observed only when DCIR-Fc constructs were devoid of glycans in their CRD.

Since we noticed an increased binding of DCIR-Fc Lec8 and DCIR_N185Q_-Fc to immobilized Lewis^b^ and Man_3_, we hypothesized that DCIR expressed on CHO Lec8 cells or DCIR_N185Q_ expressed on either wild-type or CHO Lec8 cells might be able to bind glycan-coated beads or neoglycoconjugates as well. We therefore made DCIR and DCIR_N185Q_ expressing CHO and CHO Lec8 cell lines, and analyzed them for glycan binding. FACS analysis showed similar expression levels of DCIR on all cell lines (Figure 4C). Since at low glycan concentrations binding of Lewis^b^ to DCIR was superior to binding of Man_3_ (Figure 1B), we next tested the binding of Lewis^b^-coated fluorescent beads to the different DCIR expressing cell lines. Unexpectedly, attachment of glycans to CHO Lec8-DCIR cells and CHO-DCIR_N185Q_ cells was not observed, while Lewis^b^-coated fluorescent beads strongly bound the DC-SIGN expressing cell line (Figure 4D). Binding of CHO-DC-SIGN to Lewis^b^ coated beads varied between 49 and 70% in the independent experiments, while cellular DCIR binding to Lewis^b^ coated beads was never more than 15% and never significantly higher than binding of control beads. Furthermore, also the PAA-glycans (as used in the glycan binding assay (Figure 4B)) pre-complexed with streptavidin-Alexa Fluor 647 did not show any binding to DCIR-expressing cells, while high binding was detected to DC-SIGN expressing cells (Figure 4E). GalNAc-coated fluorescent beads and GalNAc-PAA were used as negative controls. Since Hsu *et al*. [22] demonstrated DCIR binding to sulfated glycans, we additionally investigated DCIR binding to sulfo-Lewis^a^. Similar results were observed for sulfo-Lewis^a^ compared to Lewis^b^ (Figure S2), whereby immobilized glycosylated DCIR-Fc could interact with sulfo-Lewis^a^ (Figure S2A), whereas only DCIR-Fc produced in CHO Lec8 bound the immobilized sulfo-Lewis^a^ (Figure S2B). Attachment of sulfo-Lewis^a^ to CHO Lec8-DCIR cells and CHO-DCIR_N185Q_ cells was again not observed (Figure S2C and S2D). These findings demonstrate that purified DCIR binding to glycans is enhanced when DCIR carries short glycans or is unglycosylated; however, these modifications do not have any effects on the binding activity of cellular DCIR.

### Ligand Binding Decreases the Phosphorylation of The Itim in Dcir

**Figure 5:**
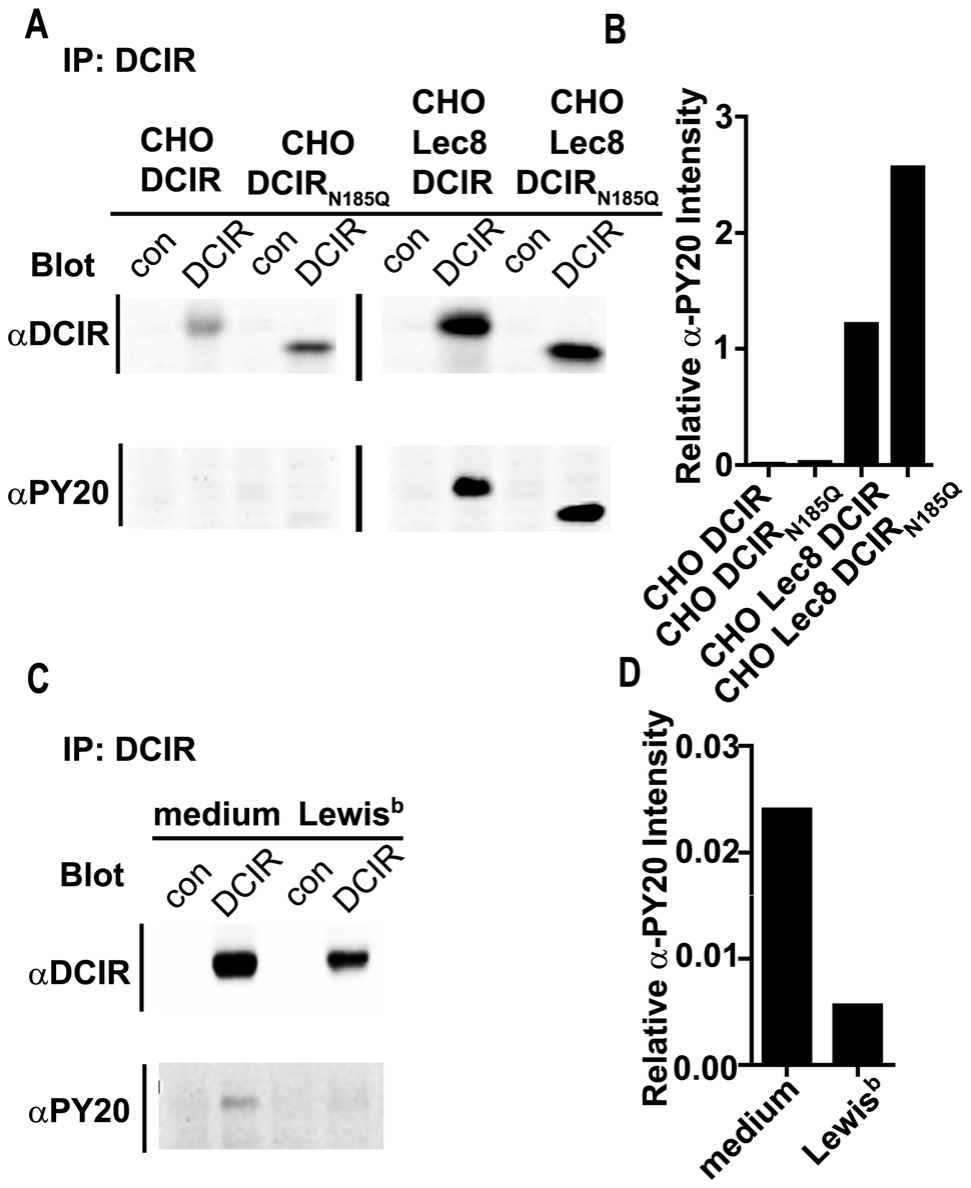
DCIR ligand binding results in decreased phosphorylation of the ITIM in DCIR. (A) DCIR phosphorylation is decreased in CHO-DCIR cells. Whole cell lysates from DCIR expressing cells were prepared and subjected to immunoprecipitation with α-DCIR (DCIR) or α-Langerin (con, control). Blots were stained for DCIR and for phosphorylated tyrosine (PY20). (B) The relative α-PY20 intensity is calculated by dividing the raw intensity of α-PY20-specific signal in the DCIR immunoprecipitations by the raw intensity of the α-DCIR-specific signal from the corresponding DCIR immunoprecipitations. One experiment out of two independent experiments is shown. (C) DCIR ligand binding results in decreased phosphorylation of the ITIM in DCIR. CHO Lec8-DCIR cells were stimulated with Lewis^b^. Whole cell lysates were prepared and subjected to immunoprecipitation with α-DCIR (DCIR) or α-Langerin (con). Blots were stained for DCIR and for phosphorylated tyrosine (PY20). (D) The relative α-PY20 intensity is calculated by dividing the raw intensity of the α-PY20-specific signal in the DCIR immunoprecipitations by the raw intensity of the α-DCIR-specific signal from the corresponding DCIR immunoprecipitations. One representative experiment out of three independent experiments is shown.

One of the interesting features of DCIR is the presence of an ITIM in its cytoplasmic domain. This motif is functional, since phosphorylation of the ITIM results in binding to the phosphatase SHP-1 [8]. Because the CRD of DCIR might be occupied with glycans, either present on the *N*-glycosylation site of the CRD or via *cis* binding to glycans on neighboring glycoproteins, signaling via this ITIM could potentially occur in resting conditions. To investigate if the presence of glycans on cells might trigger DCIR signaling via its ITIM, we investigated the steady-state phosphorylation status of the ITIM in the different DCIR expressing cell lines. DCIR was immunoprecipitated from the different cell lines and resolved by SDS-PAGE. Proteins were transferred to a nitrocellulose membrane and stained for DCIR or for phosphorylation of the ITIM, making use of a phospho-tyrosine specific antibody, thereby staining the only intracellular tyrosine residue, localized in the ITIM in DCIR. Immunoprecipitation of DCIR revealed a decrease in apparent molecular weight for the glycosylation mutant, while this was not evident for wild-type DCIR expressed in CHO Lec8 cells (Figure 5A). Interestingly, the phosphorylation status of the ITIM in DCIR was increased in CHO Lec8 cells compared to CHO cells, which expressed DCIR ligands. This observation suggests that DCIR in CHO cells becomes dephosphorylated upon ligand binding. Indeed, quantification of the DCIR and phosphorylated tyrosine signals revealed a decrease in phosphorylation of DCIR in CHO cells compared to CHO Lec8 cells (Figure 5B). Moreover, the phosphorylation of DCIR was also increased in the glycosylation mutant of DCIR in the CHO Lec8 cells, suggesting that the truncated glycans present in the CRD of DCIR in the CHO Lec8-DCIR cells trigger signaling via the ITIM in DCIR. The complete absence of glycans in the CRD of DCIR_N185Q_ in CHO Lec8-DCIR resulted in an enhanced phosphorylation of the ITIM in DCIR.

To further strengthen the finding that glycan interactions can affect the phosphorylation of DCIR, we analyzed the phosphorylation status after addition of the glycan ligands of DCIR. As DCIR expressed in CHO cells was dephosphorylated, due to potential glycan interaction, we postulated that addition of Lewis^b^, a DCIR ligand, to CHO Lec8-DCIR cells would lead to dephosphorylation of DCIR. Indeed we observed that DCIR became dephosphorylated after Lewis^b^ stimulation, confirming that Lewis^b^ is a ligand for cellular DCIR as well (Figure 5C&D). Similar results were obtained for Man_3_ (data not shown).

Our results demonstrate that DCIR binding to Lewis^b^ and Man_3_ is influenced by the glycosylation state of the CRD of DCIR. Moreover, the glycosylation of DCIR expressing cells affects the signaling function through its ITIM.

## Discussion

In this study we have shown DCIR signaling after ligand binding and the involvement of the glycosylation of DCIR in its capacity to interact with its ligands. Distinct from other CLRs, we noticed a lack of binding of DCIR ligands to cellular DCIR. This led us to the hypothesis that cellular DCIR might be masked, either by *cis* binding to glycans present on adjacent proteins; or by glycans attached to the predicted *N*-glycosylation site in the CRD of DCIR (N_185_). Glycans present on this glycosylation site can result in *cis* interactions; however they may also cause steric hindrance. Through the use of differentially glycosylated purified DCIR-Fc constructs, we observed that a decrease in glycosylation resulted in an increased binding affinity of the DCIR-Fc construct to DCIR binding glycans, illustrating the importance of the glycosylated status of DCIR for its potential to interact with glycans. A similar regulatory mechanism has also been reported for the MR, regulating both mannose recognition as well as receptor oligomerization [39].

Lee *et al*. [21] described binding of purified human DCIR to mannose and fucose monosaccharides. Although the oligosaccharide Lewis^b^ contains a fucose moiety, probably the orientation of this fucose inside Lewis^b^ is crucial for binding to DCIR, as we did not detect binding of DCIR to fucose alone. The superior binding seen to Man_3_ compared to the monosaccharide mannose might be explained by the increased multivalency of the mannose residues in Man_3_. The positive effect of multivalency on DCIR binding is further illustrated by the ability of glycosylated immobilized DCIR-Fc to bind soluble glycans and the binding of glycosylated DCIR-Fc coupled beads to CHO cells, where in both cases DCIR avidity is enhanced over the soluble DCIR-Fc construct used in the glycan binding assay. Nevertheless, although the DCIR avidity is likewise enhanced for cellular DCIR, we could not detect glycan attachment to cellular DCIR. The DCIR-Fc construct that Lee *et al*. [21] used, was produced in a FreeStyle 293 Expression System, which likely resulted in less DCIR binding glycans present on their DCIR-Fc construct, comparable to our DCIR-Fc Lec8 construct, resulting in detectable binding of soluble DCIR-Fc to immobilized glycans. Preliminary data from our laboratory confirmed that binding of DCIR-Fc to 293 cells is indeed very low (data not shown). The binding to monosaccharides that Lee *et al*. [(20)] observed could be explained by the scaffold used (BSA) or the amount of sugar moieties attached, which is different from our PAA-glycans. However, the binding signal of DCIR-Fc to glycans in both our assay and the assay reported by Lee *et al*. [21] was weak, indicating a low ligand binding capacity for DCIR.

Cellular DCIR binding to *trans* glycans is potentially prevented by the *cis* interactions with glycans present on adjacent proteins. This has been demonstrated for siglecs, which are masked by sialylated *cis*-glycans and thereby loose the ability to bind low affinity ligands [23]. Therefore, we investigated the glycan binding capacity of DCIR expressed on different cell types, which contain different glycosylation profiles. Nevertheless, glycan binding to cellular DCIR could not be observed. The high concentrations of DCIR-Fc constructs needed in the glycan binding assay suggest that DCIR clustering is necessary for optimal binding of DCIR to its ligands. Although an enhanced clustering of cellular DCIR over purified DCIR might be expected, this could be absent or altered in DCIR-transduced CHO and CHO Lec8 cell lines, compared to DCIR expressed by immune cells. In addition, the binding strength of DCIR-Fc to its glycans is low. Interactions with higher affinity ligands or increased clustering of DCIR on the cell membrane could result in detectable ligand binding, like observed for hepatitis C virus [12]. Investigating the DCIR-specific glycan binding to naturally expressing DCIR cells is difficult, due to the presence of other glycan binding receptors that share specificity for Lewis^b^ and Man_3_, like DC-SIGN. Furthermore, with the use of glycosylation mutant cells, we could elucidate the effect of mutating the DCIR *N*-glycosylation site and reducing overall cell glycosylation on DCIR-glycan interactions. We cannot completely rule out that the glycan specificity of DCIR-Fc could be distinct from naturally expressed DCIR. Nevertheless, differences herein are not observed for other CLRs, like MGL [17] and DC-SIGN [19]. In addition, we demonstrate identical DCIR specificities for three differentially glycosylated DCIR-Fc constructs (DCIR-Fc, DCIR_n185q_-Fc and DCIR-Fc Lec8), indicating that an altered DCIR glycosylation has no effect on DCIR specificity and only affects binding strength.

Another interesting feature of DCIR is the presence of an ITIM in the intracellular domain. This ITIM has been shown to be functional in both human and murine DCIR. Phosphorylation of the ITIM results in the attraction of phosphatases [8], [9]. DCIR has been shown to down modulate TLR and B cell receptor signaling [13]–[15]. However, in all cases the functionality of DCIR was assessed using monoclonal DCIR antibodies or chimeric proteins, which are far from physiological conditions and probably involve high affinity interactions.

CHO cells express oligomannosidic structures [37], which are a potential ligand for DCIR, as we showed here for Man_3_. DCIR expressed on CHO cells can therefore be masked and possibly even stimulated under steady-state conditions. Triggering by *cis* ligands has been shown indirectly for CD22 on B cells, which contains an ITIM in its intracellular tail as well. B cells with unmasked CD22 displayed a matured phenotype, suggesting that the interaction of CD22 in *cis* could result in inhibition of maturation [40]. We demonstrated that the presence of DCIR ligands on the cell surface resulted in a decreased phosphorylation of the ITIM in DCIR. DCIR is phosphorylated in the absence of DCIR ligands, as we show for DCIR expressed on the CHO Lec8 cell line. Furthermore, Lewis^b^ stimulation of CHO Lec8-DCIR cells resulted in a decreased phosphorylation of the ITIM in DCIR, confirming Lewis^b^ as DCIR ligand. The detectable Lewis^b^-induced DCIR signaling is in contrast to the absence of visible Lewis^b^ attachment to cellular DCIR. We postulate that this phenomenon might be explained by a high off-rate of DCIR ligands for the DCIR receptor. In our flow cytometric assays this high off-rate of DCIR ligands will result in the loss of attached glycans during the washing steps. In contrast, DCIR signaling is detected in cells lysed in the presence of DCIR-binding glycans.

Generally ITIMs become phosphorylated upon ligand binding. Remarkably, we observed the opposite effect. Ligand binding and the presence of DCIR ligands on the cell surface resulted in decreased phosphorylation of the ITIM in DCIR. Similar results have been demonstrated for the receptor SIRPα [41], [42]. SIRPα is a member of the immunoglobulin superfamily and contains four putative ITIMs in its intracellular tail. CD47 is a ligand for SIRPα and stimulation of SIRPα with CD47 resulted in a decreased phosphorylation [41]. Furthermore, CD47 release in the injured kidney had a similar effect on the phosphorylation status of SIRPα [42], [43]. This might be explained by compartmentalization of receptor molecules in areas lacking phosphatases under resting conditions. Receptor triggering could result in receptor mobilization to areas rich in phosphatases, thereby decreasing the phosphorylation state of the receptor, as seen for DCIR and SIRPα. Compartmentalization of proteins and changes upon receptor triggering have been reported for example for the B-cell receptor [44].

Phosphorylated ITIMs have the ability to interact with the phosphatases SHP-1, SHP-2 and SHIP-1 [45]. DCIR is able to interact with the phosphatases SHP-1 and SHP-2 [8], [9]. However, interactions with phosphatases do not necessarily lead to inhibition of cellular activation. FcεRI stimulation in combination with Trem-like transcript 1 (TLT1) led to an enhancement of calcium release, which was dependent on the attraction of SHP-2 [46]. An increased attraction of SHP-2 to the phosphorylated SIRPα in resting melanoma cells, resulted in an increased cell migration compared to stimulated melanoma cells that displayed a decreased phosphorylation of SIRPα and thereby a decreased attraction of SHP-2 [41]. Attraction of SHP-2 by unstimulated phosphorylated DCIR could therefore cause activation of cellular events as well. In contrast, DCIR stimulation, resulting in dephosphorylation, can have an inhibitory effect, explaining the negative regulatory function of DCIR in moDCs after TLR triggering [14], [15]

Recently a non-classical CLR, the Dendritic-cell–associated C-type lectin 2 (DCAL-2), has been described [47], [48]. This lectin lacks the classical calcium-binding site and the glycan specificity for this receptor has not been described yet. DCAL-2 carries an intracellular ITIM, which can interact with both SHP-1 and SHP-2 [48]. Zymosan stimulation in RAW264.7 cells expressing a chimeric protein consisting of the intracellular tail of DCAL-2 and the extracellular domains of Dectin-1 resulted in a decreased TNF-α production, compared to Dectin-1 stimulation alone. This effect was dependent on the intracellular ITIM, since it was abolished when the ITIM was mutated [48]. On the other hand, DCAL-2 signaling in immature moDCs led to an enhanced CCR7 expression and increased IL-6 and IL-10 production [47].

The phosphorylation status of the ITIM after ligand binding might also depend on the cell type on which the receptor is expressed. Antibody stimulation of SIRPα in neurons has been shown to increase the phosphorylation of SIRPα [49], resulting in an increased interaction of SHP-2. In contrast, antibody triggering of SIRPα in melanoma cells led to a decreased phosphorylation of SIRPα and a decreased interaction with SHP-2 [41]. DCIR is expressed on a wide variety of cell types [6], including dendritic cells, monocytes, macrophages and B cells. It would be interesting to investigate the phosphorylation status of DCIR in the different cell types in relation to the ligand stimulation. Nevertheless, due to the presence of other glycan binding receptors that share specificity for Lewis^b^ and Man_3_, one cannot fully exclude effects of ligand-induced signaling via these receptors on DCIR phosphorylation as well. Furthermore, differences in glycosylation after maturation of DCs have been demonstrated [50]. The glycosylation of resting DCs might therefore confer inhibitory signals via the ITIM in DCIR, contributing to the homeostatic control, as has been proposed for the dcir^−/−^ mice [10]. DCIR signaling might alter after maturation due to the change in glycosylation associated to DC maturation, releasing the inhibitory effect.

In conclusion, DCIR has the ability to interact with Man_3_ and Lewis^b^. Binding of DCIR to its ligands is modulated by the presence of glycans inside the CRD of DCIR. Although glycan binding to cellular DCIR could not be directly observed; we obtained indirect evidence of its occurrence by the changes in the phosphorylation of DCIR upon exposure to Man_3_ and Lewis^b^. This suggests that the main function of DCIR involves signaling via the ITIM upon ligand interaction. The contribution of DCIR to HIV infection [51], [52] could be caused by signaling via DCIR rather than by direct DCIR-dependent HIV attachment and internalization, in agreement with the DCIR-dependent signaling after stimulation with HIV [51]. Thus, the DCIR-ligand interaction is highly regulated through cell-specific glycosylation, in contrast to other CLRs with similar glycan specificities, like DC-SIGN. *Trans* interactions can therefore only occur under permissive conditions, whereas both *cis* and *trans* interactions can result in DCIR-mediated signaling. Further research should reveal how DCIR masking regulates DC biology and explore its potential implications in the therapeutic modulation of the immune response.

## Supporting Information

Figure S1 **Glycosylation of DCIR-Fc.** The apparent molecular weight of DCIR-Fc and the glycosylation mutant of DCIR-Fc (DCIR_N185Q_-Fc) was analyzed by SDS-PAGE and stained with α-DCIR. The apparent molecular weight of DCIR_N185Q_-Fc was decreased compared to DCIR-Fc, suggesting glycosylation of the wild-type DCIR-Fc construct.
(TIF)

Figure S2 **DCIR interacts with sulfo-Lewis^a^.** (A) Immobilized DCIR-Fc constructs interact with sulfo-Lewis^a^. Biotin-labeled sulfo-Lewis^a^-PAA was added at a concentration of 5 µg/ml to immobilized DCIR-Fc and DCIR-Fc Lec8. Depicted is Fc-specific binding with the EGTA control subtracted. (B) Increased binding of DCIR to sulfo-Lewis^a^ in the presence of truncated glycans on the DCIR-Fc construct. Binding of DCIR-Fc and DCIR-Fc Lec8 to immobilized sulfo-Lewis^a^-PAA was measured in a glycan binding assay. Average ± SD of duplicates is shown. (C) Binding of sulfo-Lewis^a^-coated beads to cellular DCIR could not be detected. Binding of fluorescent labeled sulfo-Lewis^a^-coated beads to the different DCIR expressing cell lines was measured by flow cytometry in the absence of serum. As a control binding of the fluorescent labeled sulfo-Lewis^a^-coated beads to CHO-DC-SIGN was measured. (D) Glycan binding of sulfo-Lewis^a^-PAA to cellular DCIR could not be detected. Binding of sulfo-Lewis^a^-PAA pre-incubated with streptavidin-Alexa Fluor 647 to the different DCIR and DC-SIGN expressing cell lines was measured by flow cytometry after 2 hours incubation at 37°C. Binding of biotinylated sulfo-Lewis^a^-PAA to CHO-DC-SIGN served as a positive control.
(TIF)
