# Supplementary figures and images for: Ligand Binding and Signaling of Dendritic Cell Immunoreceptor (DCIR) Is Modulated by the Glycosylation of the Carbohydrate Recognition Domain
Source: PLoS One. 2013 Jun 11;8(6):e66266. doi: 10.1371/journal.pone.0066266 (PMC3679074; doi:10.1371/journal.pone.0066266)

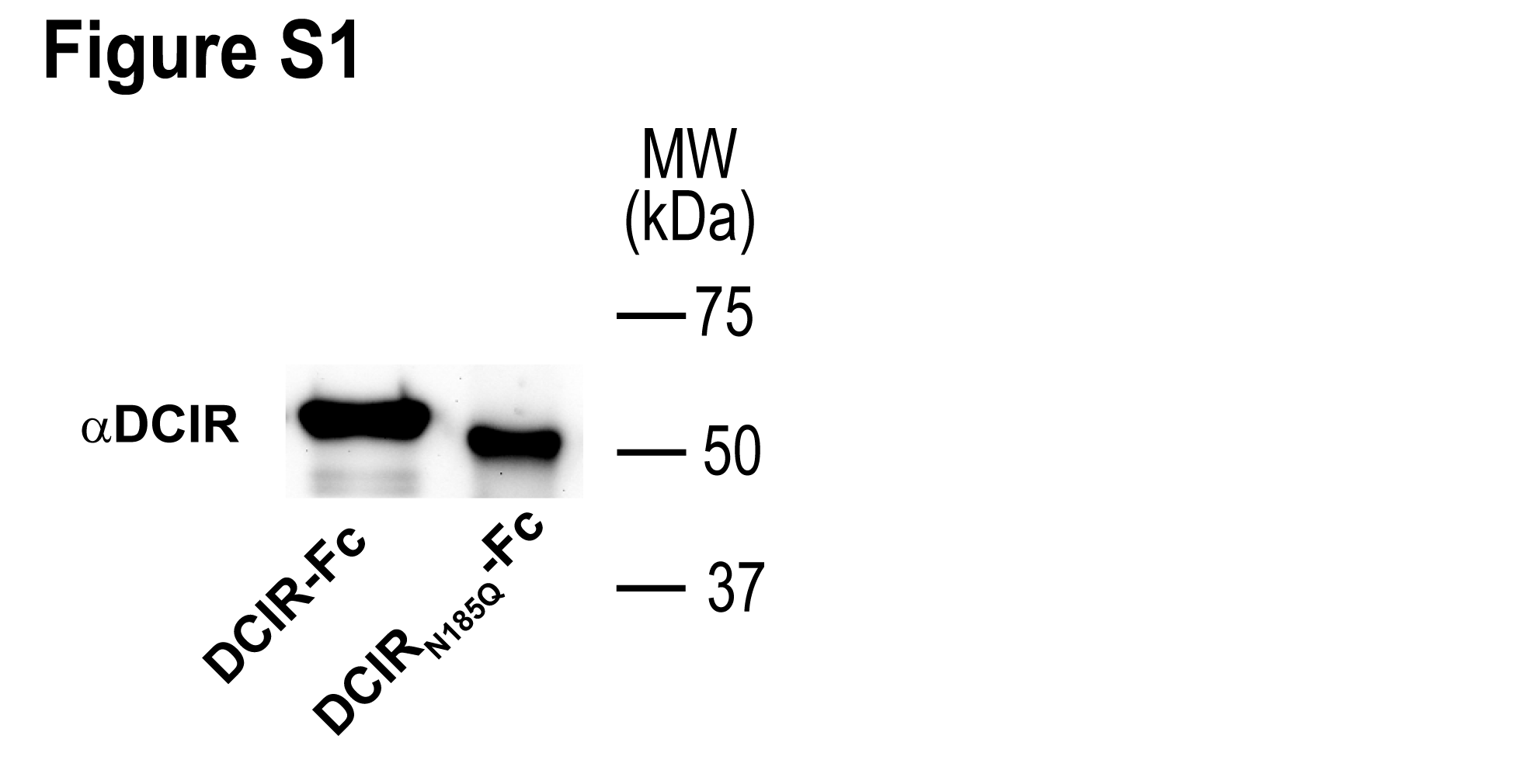

Supplement: Figure S1 — Glycosylation of DCIR-Fc. The apparent molecular weight of DCIR-Fc and the glycosylation mutant of DCIR-Fc (DCIRN185Q-Fc) was analyzed by SDS-PAGE and stained with α-DCIR. The apparent molecular weight of DCIRN185Q-Fc was decreased compared to DCIR-Fc, suggesting glycosylation of the wild-type DCIR-Fc construct. (TIF) [file pone.0066266.s001.tif]

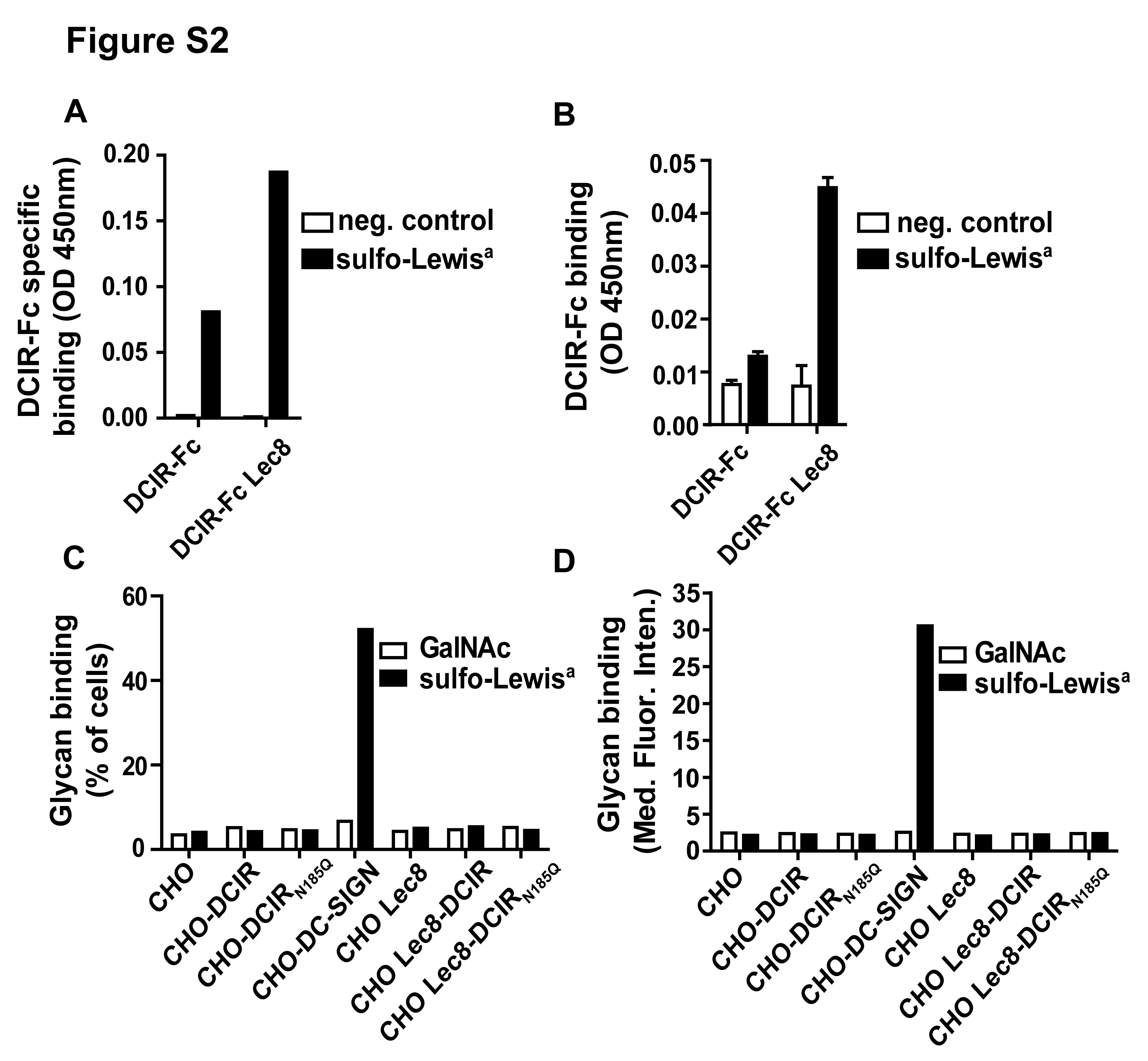

Supplement: Figure S2 — DCIR interacts with sulfo-Lewisa. (A) Immobilized DCIR-Fc constructs interact with sulfo-Lewisa. Biotin-labeled sulfo-Lewisa-PAA was added at a concentration of 5 µg/ml to immobilized DCIR-Fc and DCIR-Fc Lec8. Depicted is Fc-specific binding with the EGTA control subtracted. (B) Increased binding of DCIR to sulfo-Lewisa in the presence of truncated glycans on the DCIR-Fc construct. Binding of DCIR-Fc and DCIR-Fc Lec8 to immobilized sulfo-Lewisa-PAA was measured in a glycan binding assay. Average ± SD of duplicates is shown. (C) Binding of sulfo-Lewisa-coated beads to cellular DCIR could not be detected. Binding of fluorescent labeled sulfo-Lewisa-coated beads to the different DCIR expressing cell lines was measured by flow cytometry in the absence of serum. As a control binding of the fluorescent labeled sulfo-Lewisa-coated beads to CHO-DC-SIGN was measured. (D) Glycan binding of sulfo-Lewisa-PAA to cellular DCIR could not be detected. Binding of sulfo-Lewisa-PAA pre-incubated with streptavidin-Alexa Fluor 647 to the different DCIR and DC-SIGN expressing cell lines was measured by flow cytometry after 2 hours incubation at 37°C. Binding of biotinylated sulfo-Lewisa-PAA to CHO-DC-SIGN served as a positive control. (TIF) [file pone.0066266.s002.tif]
